# *DLGAP5* Regulates the Proliferation, Migration, Invasion, and Cell Cycle of Breast Cancer Cells via the JAK2/STAT3 Signaling Axis

**DOI:** 10.3390/ijms242115819

**Published:** 2023-10-31

**Authors:** Yujie Li, Jie Wei, Yao Sun, Wenqian Zhou, Xiaoya Ma, Jinping Guo, Huan Zhang, Tianbo Jin

**Affiliations:** 1 Key Laboratory of Resource Biology and Biotechnology in Western China, Ministry of Education, School of Life Sciences, Northwest University, Xi’an 710069, China; liyujie@stumail.nwu.edu.cn (Y.L.); weijie@stumail.nwu.edu.cn (J.W.); sunyao@stumail.nwu.edu.cn (Y.S.); zhouwenqian@stumail.nwu.edu.cn (W.Z.); maxiaoya@stumail.nwu.edu.cn (X.M.); guojinping@stumail.nwu.edu.cn (J.G.); zhanghuan@stumail.nwu.edu.cn (H.Z.); 2 College of Life Science, Northwest University, Xi’an 710127, China; 3 Provincial Key Laboratory of Biotechnology of Shaanxi Province, Northwest University, Xi’an 710069, China

**Keywords:** breast cancer, bioinformatics, *DLGAP5*, JAK2/STAT3 signaling pathway, proliferation, migration, invasion, cell cycle

## Abstract

The aim of this study was to discover new biomarkers to detect breast cancer (BC), which is an aggressive cancer with a high mortality rate. In this study, bioinformatic analyses (differential analysis, weighted gene co-expression network analysis, and machine learning) were performed to identify potential candidate genes for BC to study their molecular mechanisms. Furthermore, Quantitative Real-time PCR and immunohistochemistry assays were used to examine the protein and mRNA expression levels of a particular candidate gene (*DLGAP5*). And the effects of *DLGAP5* on cell proliferation, migration, invasion, and cell cycle were further assessed using the Cell Counting Kit-8 assay, colony formation, Transwell, wound healing, and flow cytometry assays. Moreover, the changes in the JAK2/STAT3 signaling-pathway-related proteins were detected by Western Blot. A total of 44 overlapping genes were obtained by differential analysis and weighted gene co-expression network analysis, of which 25 genes were found in the most tightly connected cluster. Finally, *NEK2*, *CKS2*, *UHRF1*, *DLGAP5*, and *FAM83D* were considered as potential biomarkers of BC. Moreover, *DLGAP5* was highly expressed in BC. The down-regulation of *DLGAP5* may inhibit the proliferation, migration, invasion, and cell cycle of BC cells, and the opposite was true for *DLGAP5* overexpression. Correspondingly, silencing or overexpression of the *DLGAP5* gene inhibited or activated the JAK2/STAT3 signaling pathway, respectively. *DLGAP5*, as a potential biomarker of BC, may impact the cell proliferation, migration, invasion, cell cycle, and BC development by modulating the JAK2/STAT3 signaling pathway.

## 1. Introduction

Breast cancer (BC) is an aggressive cancer and a leading cause of cancer-associated death in women worldwide. Current global cancer burden data estimate 260,000 BC cases in the last two years [1]. In China, more than three million women are diagnosed with BC each year. Of these cases, approximately 70–80% of patients with early non-metastatic BC are cured, while late-stage BC with distant organ metastasis is currently considered untreatable by available therapies [2]. Studies have revealed marked disparities in mortality rates of BC among different countries, where developed countries exhibited significantly lower rates compared to underdeveloped regions. This phenomenon could primarily be attributed to a combination of early detection strategies, timely diagnosis, and improved access to effective treatments [1,3]. Currently, the traditional approach to detecting BC involved screening of blood tumor markers in conjunction with X-ray and ultrasound imaging, resulting in an accuracy rate of over 80% [4,5]. Nonetheless, early diagnosis of BC remains a pressing issue in underdeveloped regions without access to advanced diagnostic and therapeutic methods. Thus, the discovery of potential biomarkers as indicators for BC has been crucial in decreasing its incidence and enhancing the efficacy of treatment.

Bioinformatics, once one of the newest areas of biological research, has been widely used to search for potential biomarkers of tumors. Chen et al. have applied bioinformatics to identify 11 genes (including *FN1*, *AUKRA*, and *CCNB1*) as potential prognostic biomarkers for BC [6]. Weighted gene co-expression network analysis (WGCNA) is a popular gene–phenotype relationships analysis that is able to analyze complex relationships between genes and phenotypes [7]. Machine learning has matured in bioinformatics applications to identify potential risk factors, mechanisms, prospective biomarkers, and therapeutic targets for a variety of diseases [8]. Currently, numerous studies have combined WGCNA and machine learning to screen for potential cancer markers. Su et al. [9] have screened for genes associated with colon cancer based on machine learning and WGCNA. Chen et al. [10] identified the key prognostic genes of triple-negative BC based on LASSO and WGCNA. As a result, bioinformatics has become particularly essential for cancer research.

In this study, the potential biomarker of BC (*DLGAP5*) was screened by bioinformatic methods (differential analysis, machine learning, WGCNA). The expression levels of *DLGAP5* and its effects on BC cell functions (invasion, migration, proliferation, and cycle) in BC were examined by experiments such as Western Blot (WB), Quantitative Real-time PCR (qRT-PCR), Cell Counting Kit-8 assay (CCK8), Transwell, wound-healing assay, colony formation assay, and flow cytometry assays. To further investigate the molecular mechanism of *DLGAP5*, KEGG enrichment analysis of *DLGAP5* and its related genes was performed through the DAVID database (https://david.ncifcrf.gov/ (accessed on 11 May 2023)), and it was found that *DLGAP5* might play a role in the JAK/STAT pathway. Later, WB and qRT-PCR were used to detect the JAK2/STAT3 pathway proteins to further explore the relationship between *DLGAP5* and the JAK/STAT pathway. This study may provide new ideas and directions for the early diagnosis and treatment of BC.

## 2. Results

### 2.1. Identification of DEGs in BC Based on Bioinformatic Methods

**Figure 1:**
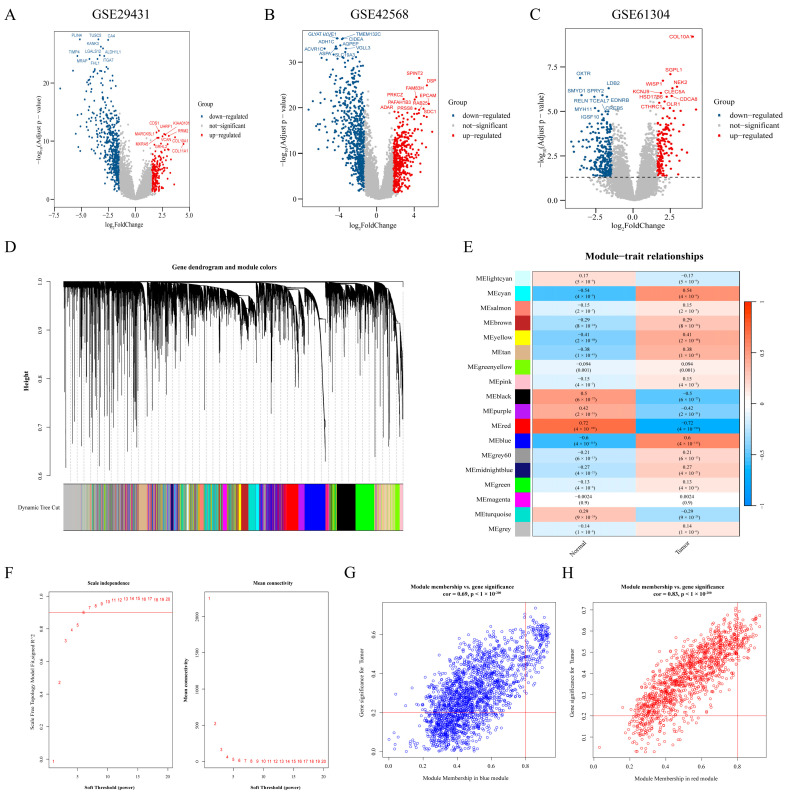
Identification of DEGs and the determination of the optimal soft threshold of scale-free topology and the hub genes in the key BC-related modules. (**A**–**C**) Volcano plot of DEGs in the GSE29431, GSE42568, and GSE61304 datasets. Red dots represent up-regulated genes and blue dots represent down-regulated genes. (**D**) Dendrogram of all genes with dissimilarity clustered on topological overlap. (**E**) Correlation of eighteen modules with two traits (control and BC). The red (R = −0.72, *p* < 0.001) and blue modules (R = 0.6, *p* < 0.001) were significantly correlated with BC. (**F**) The scale-free topology fit index (R^2^) and the mean connectivity (k) for soft-threshold power (β) from 1 to 20. The red number indicated the soft threshold (β) from 1 to 20. (**G**,**H**) Scatterplot of Module Membership (MM) and gene significance (GS) in the red and blue modules of BC.

The differences were analyzed for the GSE29431, GSE42568, and GSE61304 datasets. Under the threshold (|log_2_FC| > 1.5 and *p* < 0.05), the 703 DEGs were screened from GSE29431, of which 193 were up-regulated genes and 510 were down-regulated genes. The 1145 DEGs were obtained from GSE42568 (463 up-regulated genes and 682 down-regulated genes). A total of 417 DEGs were screened by GSE61304, of which 152 were up-regulated and 265 were down-regulated. The volcanoes of DEGs were then plotted according to log_2_FC and −log_10_ (adjust *p*) (Figure 1A–C). 

### 2.2. Screening BC-Related Genes through WGCNA

A gene co-expression network was constructed using WGCNA to identify BC-related module genes in The Cancer Genome Atlas (TCGA) dataset. At first, the similarity matrix was calculated, and then converted to the adjacency matrix by optimal soft threshold (β = 6, R^2^ = 0.9) (Figure 1F). Next, the TOM of the adjacency matrix was converted to dissTOM (dissTOM = 1 − TOM) for hierarchical clustering. The dynamic shear tree clustered similarity genes based on topological overlap and these genes were then divided into multiple modules (Figure 1D). The correlation between module genes and clinical traits was further investigated, and genes associated with BC were acquired in strongly correlated modules (absolute value of correlation ≥0.6), as illustrated in Figure 1E. It was ultimately determined that the red module was strongly negatively correlated with BC (R = −0.72, *p* < 0.001) and the blue module was positively related to BC (R = 0.60, *p* < 0.001). Critical genes in the modules were selected according to GS > 0.2 and MM > 0.8. Last, a total of 73 and 153 pivotal genes associated with BC were identified in the red and blue modules, respectively (Figure 1G,H).

### 2.3. PPI Network Construction of Intersection Genes

**Figure 2:**
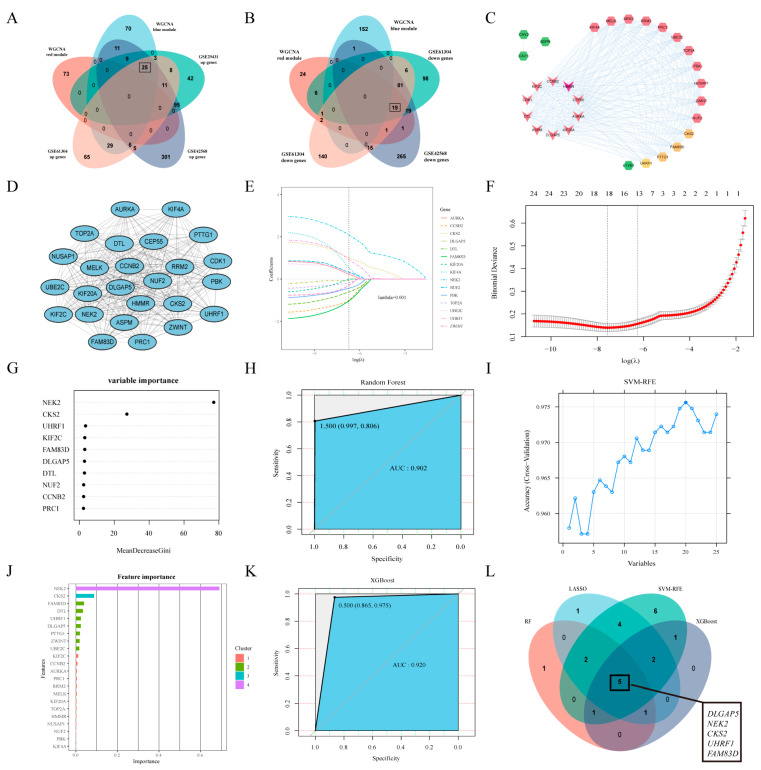
Construction PPI network and hub gene screening. (**A**,**B**) Venn diagram of 25 intersection genes between differentially up-regulated and down-regulated genes in GSE29431, GSE42568, and GSE61304 datasets and hub genes in the red and blue modules. (**C**) PPI network of 44 intersecting genes; the darker the color, the more node genes connected. (**D**) Tight-knit clusters in PPI networks screened by MCODE plug-in of Cytoscape. (**E**) The coefficients of 15 feature genes shown by log(λ). (**F**) The relationship between binomial deviation and log(λ) by 10-fold cross-validation (CV). (**G**) The mean decrease Gini coefficients of genes in the RF classifier. It shows the top 10 most important genes. (**H**) The Receiver Operating Characteristic (ROC) curve of the RF classifier (area under the curve (AUC) = 0.902). (**I**) The accuracy of curve changes after SVM-RFE cross verification. (**J**) Ranked feature importance of XGBoost algorithm. (**K**) The ROC curve of the XGBoost classifier (AUC = 0.920). (**L**) Intersection genes of four machine learning algorithms (*DLGAP5*, *NEK2*, *CKS2*, *UHRF1*, and *FAM83D*).

When the up-regulated and down-regulated DEGs of BC vs. Normal were separately intersected with the gene members of the two key gene modules (red and blue module), 25 and 19 intersection genes were identified, respectively (Figure 2A,B). The construction of the PPI network for 44 intersection genes was conducted through the STRING database (https://string-db.org/ (accessed on 12 May 2023)) and visualized by Cytoscape (Version 3.9.1), which had 29 nodes and 301 edges after removing the unconnected nodes (Figure 2C).

### 2.4. Key Genes Screening in BC

The MCODE (Cytoscape’s plug-in, Version 2.0.2) was selected for the most tightly bound cluster of PPI network (Figure 2D), which had 25 genes and 297 edges. Four machine learning algorithms were used to narrow down the feature genes, starting with a Lasso analysis on the TCGA dataset. As shown in Figure 2E, with an optimal log(λ) of 0.001, 15 genes were retained from 25 characterized genes after Lasso analysis. The minimum binomial deviation from the optimal log(λ) was observed by 10-fold cross-validation, indicating that these 15 genes were optimally selected by Lasso (Figure 2F). In addition, with the RF and XGBoost algorithm, the top 10 genes were screened from 25 genes, separately, and they also displayed promising generalization performance (Figure 2G,H,J,K). The SVM-RFE analysis revealed that the SVM model based on 20 feature genes showed the highest accuracy (Figure 2I). The genes gained from the four machine learning algorithms were intersected, and ultimately *DLGAP5*, *NEK2*, *CKS2*, *UHRF1*, and *FAM83D* were identified as potential genes in BC development (Figure 2L).

### 2.5. Differential Expression, Survival Analysis, and Diagnostic Accuracy Analysis of Key Genes

**Figure 3:**
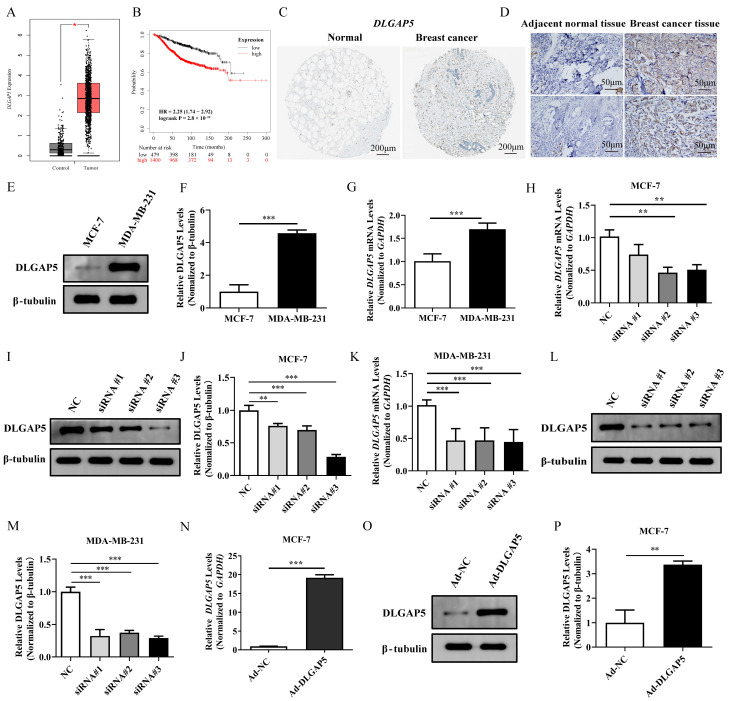
Expression of *DLGAP5* and verification of *DLGAP5* knockdown and overexpression efficiency. (**A**) The expression level of *DLGAP5* was verified by the GEPIA database (* *p* < 0.05). (**B**) Survival analysis of *DLGAP5* was performed using the Kaplan–Meier plotter database. (**C**) IHC of DLGAP5 in BC tissues and normal breast tissues in the HPA database. (**D**) IHC staining of DLGAP5 protein in BC and paracancer tissues collected from seven cases. (**E**,**G**) The relative expression of *DLGAP5* in two cell lines detected by qRT-PCR and WB (*** *p* < 0.001). (**F**) Statistics on (**E**) gray value (*** *p* < 0.001). (**H**,**I**,**K**,**L**) The knockdown efficiency of three interference sequences on *DLGAP5* in MCF-7 and MDA-MB-231 cells detected by qRT-PCR and WB (** *p* < 0.01, *** *p* < 0.001). (**J**,**M**) Statistics on (**I**,**L**) gray value (** *p* < 0.01, *** *p* < 0.001). (**N**,**O**) Detection of *DLGAP5* overexpression efficiency in MCF-7 cell via qRT-PCR and WB (*** *p* < 0.001). (**P**) The gray value analysis of (**O**) (** *p* < 0.01).

To test whether *NEK2*, *CKS2*, *UHRF1*, *DLGAP5*, and *FAM83D* could be used as diagnostic biomarkers for BC, their expression levels, survival rate and diagnostic ability were evaluated. *NEK2*, *CKS2*, *UHRF1*, *DLGAP5*, and *FAM83D* were highly expressed in the training group (GSE29431 and GSE42568) and the test group (GSE52604, GSE65194, and GSE27562) with significant differences (*p* < 0.05, Supplementary Figure S2). The expression was further verified in the Gene Expression Profiling Interactive Analysis (GEPIA, http://gepia.cancer-pku.cn/ (accessed on 21 March 2023)) database and the results were consistent with the above (Supplementary Figure S1E–H and Figure 3A). Furthermore, the IHC results obtained from the Human Protein Atlas database (HPA, https://www.proteinatlas.org/ (accessed on 25 March 2023)) also indicated that the expression of *CKS2*, *UHRF1*, and *DLGAP5* was higher in BC tissues than in normal breast tissues (Supplementary Figure S1I–K and Figure 3C).

To further explore the clinical value of the underlying genes, overall survival analyses were performed using the Kaplan–Meier plotter database (https://kmplot.com/analysis/ (accessed on 10 March 2023)). And patients with BC were divided into high and low expression groups based on the expression levels of *NEK2*, *CKS2*, *UHRF1*, *DLGAP5*, and *FAM83D* for the overall survival analysis. Risk ratios were provided and *p*-values were calculated using the log-rank test. *NEK2*, *CKS2*, *UHRF1*, *DLGAP5*, and *FAM83D* were highly expressed in BC patients with poor prognosis (Supplementary Figure S1A–D and Figure 3B). Moreover, five genes had AUC diagnostic scores above 0.82 in the training group. In GSE52604 and GSE65194, the AUC scores of these genes exceeded 0.91 (Supplementary Figure S3). These results suggested that *NEK2*, *CKS2*, *UHRF1*, *DLGAP5*, and *FAM83D* were promising diagnostic biomarkers for BC.

### 2.6. Cell Localization and Expression Verification of DLGAP5

The above studies found that *DLGAP5* was highly expressed in BC tumor cells and was associated with poor prognosis, suggesting it had a good diagnostic value. However, there is limited research on its relationship with BC. Therefore, we further investigated *DLGAP5* in the follow-up. First, an analysis of the scRNA-seq dataset (GSE176078) was performed to clarify the localization of *DLGAP5* and its expression pattern in single cells. The cells were annotated into five clusters based on the obtained marker genes: malignant cells (*ERBB2* and *BRCA1*), fibroblast cells (*PDGFRB* and *COL1A1*), endothelial cells (*PECAM1* and *MCAM*), epithelial cells (*EPCAM* and *ZEB1*), and others. By locating the *DLGAP5* expression, it was primarily expressed in malignant cells (Supplementary Figure S1L–O). Seven BC samples and seven para-cancer tissue samples were collected for IHC assays to verify the DLGAP5 expression. The results suggested that *DLGAP5* was highly expressed in BC tissues (Figure 3D). The expression of *DLGAP5* was detected using qRT-PCR and WB in the MDA-MB-231 and MCF-7 cell lines, and the protein levels were also consistent with this trend, with the DLGAP5 protein expression in MDA-MB-231 being approximately four times higher than that in MCF-7 (Figure 3E–G).

### 2.7. DLGAP5 Affected the Proliferation and Colony Formation of BC Cell Lines

**Figure 4:**
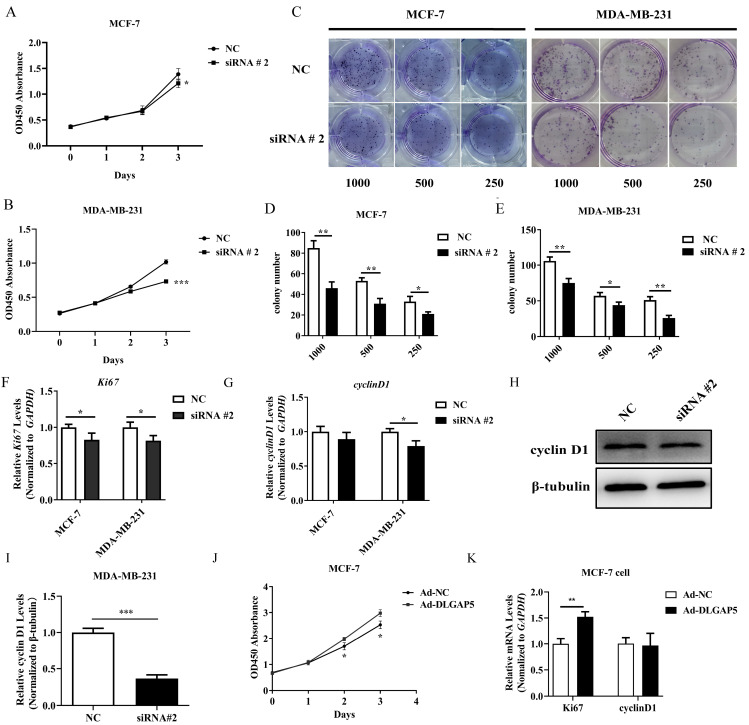
Effect of *DLGAP5* knockdown on cell proliferation and clonal formation. (**A**,**B**) Down-regulating *DLGAP5* inhibited MCF-7 and MDA-MB-231 cell proliferation (* *p* < 0.05, *** *p* < 0.001). (**C**) *DLGAP5* down-regulation inhibited the clonal formation of two cell lines. (**D**,**E**) The number of cell clones of MCF-7 and MDA-MB 231 cell lines (* *p* < 0.05, ** *p* < 0.01). (**F**,**G**) qRT-PCR was used to detect *Ki67* and *cyclin D1* gene expression in two cells when *DLGAP5* expression was low (* *p* < 0.05). (**H**) WB detected *cyclin D1* expression level in MDA-MB-231 when *DLGAP5* knockdown. (**I**) The gray value analysis of (**H**) (*** *p* < 0.001). (**J**) Overexpression of *DLGAP5* detected the proliferation ability of MCF-7 by CCK8 assay (* *p* < 0.05). (**K**) qRT-PCR detected *Ki67* and *cyclinD1* expression level in MCF-7 (** *p* < 0.01).

The NC, siRNA #1, siRNA #2, and siRNA #3 sequences of *DLGAP5* were transfected in two cell lines, and then the knockdown efficiency of *DLGAP5* was examined. The results showed that siRNA #2 and siRNA #3 interference sequences significantly reduced *DLGAP5* expression at the gene and protein levels of MCF-7 and MDA-MB-231 (Figure 3H–M). The siRNA #2 interference sequence was selected for subsequent analysis. A series of in vitro functional assays were carried out to investigate the effects of *DLGAP5* on BC cell lines. The results of the CCK8 assays suggested that *DLGAP5* silencing had decreased the proliferation ability of MDA-MB-231 and MCF-7 cell lines (Figure 4A,B). Similarly, the results of the colony formation assay reflected that knockdown of the *DLGAP5* had significantly reduced colony formation numbers and cell-independent viability compared to the NC group (Figure 4C–E). Subsequently, the expression of proliferation-related genes (*Ki67* and *cyclinD1*) was detected. More precisely, the gene expression of *Ki67* was reduced in two cell lines and *Cyclin D1* was decreased at the gene and protein levels in MDA-MB-231, whereas there was no effect in the MCF-7 cell line (Figure 4F–I).

The relative expression of *DLGAP5* in the MCF-7 cell line was low; therefore, it was chosen to verify the transfection efficiency of adenovirus overexpression of *DLGAP5* (Figure 3N–P). The gene and protein expression levels of *DLGAP5* were 20 and 6 times higher in the Ad-DLGAP5 group than the Ad-NC group, respectively. It was evident that the *DLGAP5* overexpressed adenovirus vector successfully induced high expression of *DLGAP5*. Then, the results demonstrated a significant increase in cell proliferation and the number of cell clones in the MCF-7 cell line following adenovirus transfection, as compared to Ad-NC (Figure 4J). Additionally, *Ki67* expression was markedly up-regulated while *Cyclin D1* remained unchanged (Figure 4K).

### 2.8. DLGAP5 Inhibited the Migration and Invasion of BC Cells

**Figure 5:**
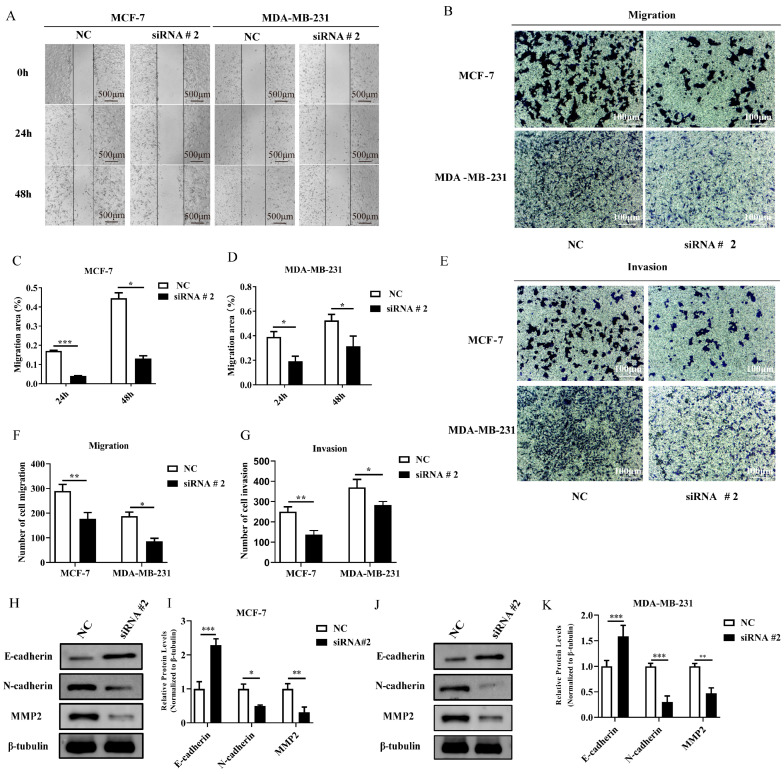
Low expression of *DLGAP5* inhibited the migration and invasion of BC cells. (**A**) Wound healing of two cell lines after down-regulating *DLGAP5*. (**C**,**D**) The wound healing statistics of MCF-7 and MDA-MB-231 (* *p* < 0.05, *** *p* < 0.001). (**B**,**E**) The migration and invasion ability of two cell lines were detected via Transwell assay after down-regulating *DLGAP5*. (**F**,**G**) The migration and invasion cell number statistics of MCF-7 and MDA-MB-231 (* *p* < 0.05, ** *p* < 0.01). (**H**,**J**) WB-detected E-cadherin, N-cadherin, and MMP2 expression levels in two cells with *DLGAP5* knockdown. (**I**,**K**) The gray value analysis of (**H**,**J**) (* *p* < 0.05, ** *p* < 0.01, *** *p* < 0.001).

Subsequently, the impact of *DLGAP5* on the migration and invasive ability of BC cells was investigated using wound-healing and Transwell assays. The results demonstrated that the knockdown of *DLGAP5* significantly attenuated the migratory and invasive potential of the MDA-MB-231 and MCF-7 cell lines (Figure 5A–G). The expression of genes related to invasion and migration was assessed using qRT-PCR and WB. The expression of metastasis-related factors E-cadherin and N-cadherin was up-regulated and down-regulated, respectively, while the expression of invasion factor MMP2 was significantly reduced in response to low *DLGAP5* expression (Figure 5H–K).

**Figure 6:**
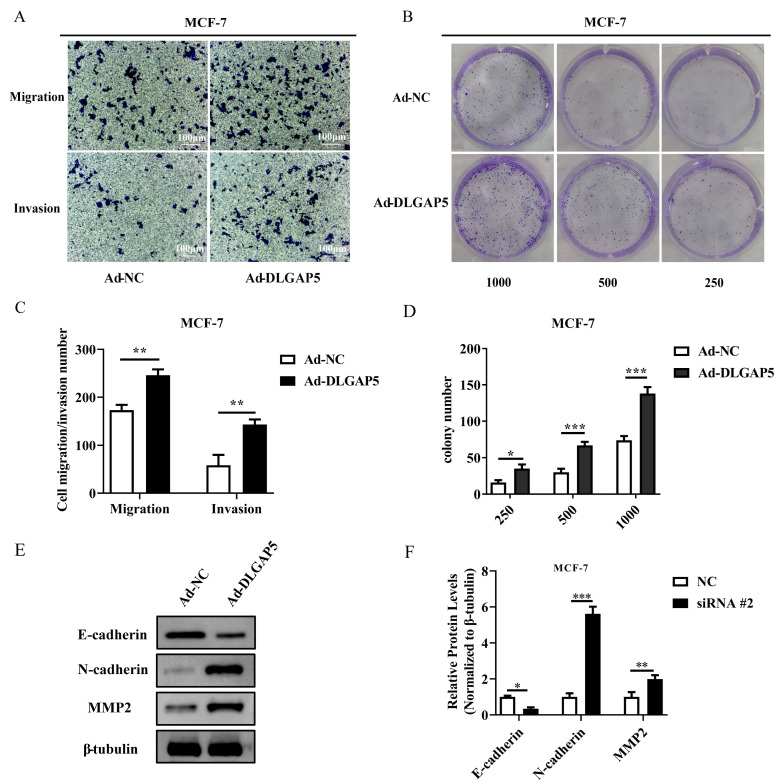
Overexpression of *DLGAP5* promoted the proliferation, migration, and invasion of BC cells. (**A**) The migration and invasion ability of MCF-7 was detected via Transwell assay after up-regulating *DLGAP5*. (**B**) Overexpression of *DLGAP5* was used to detect the clonal formation of MCF-7. (**C**,**D**) Statistics on the MCF-7 cell migration, colony, and invasion numbers (* *p* < 0.05, ** *p* < 0.01, *** *p* < 0.001). (**E**) WB-detected E-cadherin, N-cadherin, and MMP2 expression levels in MCF-7. (**F**) The gray value analysis of (**E**) (* *p* < 0.05, ** *p* < 0.01, *** *p* < 0.001).

To further verify the influence of *DLGAP5* on the migration and invasion of BC cells, we overexpressed *DLGAP5*, and then tested the migration and invasion abilities. The cell migration and invasion of Ad-*DLGAP5* was higher than that of the control group, and overexpression of *DLGAP5* could promote the migration and invasion of MCF-7 (Figure 6A–D). At the same time, E-cadherin protein was down-regulated and N-cadherin and MMP2 expression were markedly up-regulated, suggesting that overexpression of *DLGAP5* in MCF-7 promoted migration and invasion (Figure 6E,F).

### 2.9. DLGAP5 Affected the Cell Cycle of BC Cell

**Figure 7:**
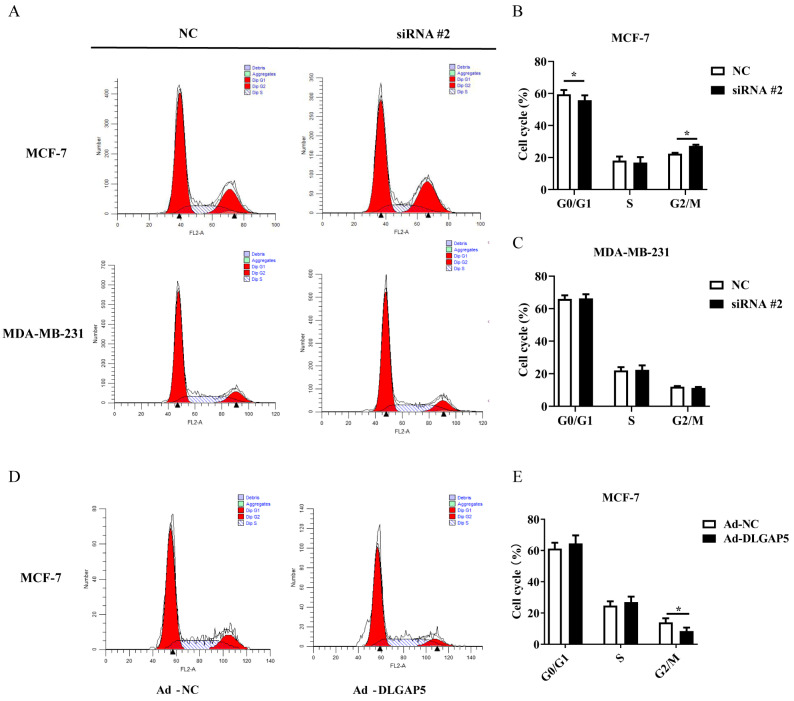
*DLGAP5* affected the cell cycle of BC cell. (**A**) The effect of down-regulation of *DLGAP5* on the cell cycle distribution of MCF-7 and MDA-MB-231. The x-coordinate represents DNA content and the y-coordinate represents the number of effective cells. The black triangle indicates the peak. (**B**,**C**) Statistics on the cycle distribution of MCF-7 and MDA-MB-231 (* *p* < 0.05). (**D**) The effect of up-regulation of *DLGAP5* on the cell cycle distribution of MCF-7. The black triangle indicates the peak. (**E**) Statistics on the cycle distribution of MCF-7 cell (* *p* < 0.05).

The PI single-stain cell cycle kit examined the effect of *DLGAP5* expression on the cycle distribution of both MCF-7 and MDA-MB-231. It was found that after knocking out the *DLGAP5* gene, the number of MCF-7 distributed in the G0/G1 and G2/M phases varied, indicating that cells were blocked during the G0 phase transition to G1 and G2 phase transition to M phase, and that the cell cycle was suppressed. However, no significant differences were found in MDA-MB-231. Then, after overexpression of *DLGAP5*, the cell cycle displayed a reduced distribution of G2/M-phase cells in the Ad-*DLGAP5* group, indicating that *DLGAP5* up-regulation accelerated MCF-7 cell cycle progression (Figure 7A–E).

### 2.10. DLGAP5 Regulated JAK2/STAT3 Signaling Pathway in BC Cell Lines

In order to further understand the mechanism of *DLGAP5* affecting BC, the 310 genes interacting with *DLGAP5* were collected from the STRING database, and the PPI network with 311 nodes and 13,303 edges was constructed (Supplementary Figure S4A). GO enrichment and KEGG pathway analyses of these genes showed that they might play the biological functions of “protein binding”, “protein kinase binding”, and “cyclin-dependent protein serine/threonine kinase regulatory activity”, and participate in the “JAK-STAT signaling pathway”, “cell cycle”, and “oocyte meiosis” pathways (Supplementary Tables S3 and S4). In addition, we analyzed the correlation between JAK-STAT signaling pathway genes obtained through the KEGG website (https://www.genome.jp/kegg/ (accessed on 27 May 2023)) and *DLGAP5* by using R (Supplementary Table S5). The genes whose absolute value of correlation was greater than 0.03 were also shown (Supplementary Figure S4B and Table S6). *DLGAP5* exhibited the highest correlation with *STAT1* (Supplementary Figure S4C–H).

**Figure 8:**
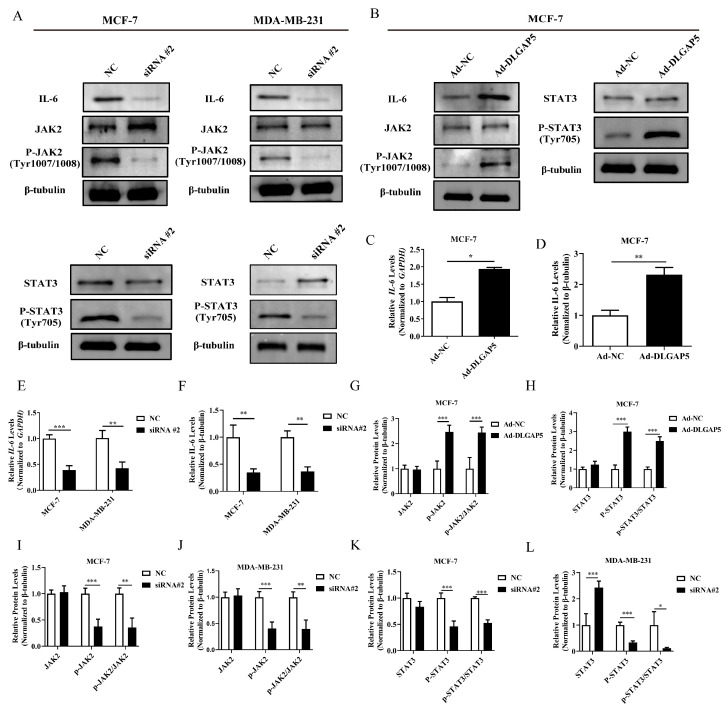
*DLGAP5* affected JAK2/STAT3 signaling pathway in BC cells. (**A**) WB was performed to detect the protein expressions of IL-6, JAK2, P-JAK2, STAT3, and P-STAT3 after MCF-7 and MDA-MB-231 were transfected with siRNA #2. (**B**) WB was performed to detect the protein expressions of IL-6, JAK2, P-JAK2, STAT3, and P-STAT3 after *DLGAP5* high expression. (**C**,**G**,**H**) Statistics on (**B**) gray value (* *p* < 0.05, *** *p* < 0.001). (**D**) qRT-PCR detected *IL-6* expression level in MCF-7 after *DLGAP5* high expression (** *p* < 0.01). (**F**) qRT-PCR detected *IL-6* expression level in MCF-7 and MDA-MB-231 after *DLGAP5* low expression (** *p* < 0.01). (**E**,**I**–**L**) Statistics on (**A**) gray value (* *p* < 0.05, ** *p* < 0.01, *** *p* < 0.001).

First, we detected the effect of *DLGAP5* on *IL-6* by WB and qRT-PCR. The results showed that when *DLGAP5* was knocked down, the gene and protein expressions of inflammatory cytokine *IL-6* were significantly reduced. Overexpression of *DLGAP5* increased the expression of *IL-6*. Subsequently, the effect of *DLGAP5* on the JAK2/STAT3 pathway was also detected. In this study, the protein levels of p-JAK2/JAK2 protein levels were also significantly reduced when *DLGAP5* expression was decreased. Simultaneously, the changes of the downstream protein STAT3 of the JAK2 pathway and its phosphorylation level were also examined. It was found that down-regulation of *DLGAP5* in MCF-7 and MDA-MB-231 cell lines dramatically decreased p-STAT3 expression and the p-STAT3/STAT3 ratio. Therefore, the p-STAT3 signaling was inhibited (Figure 8). Moreover, the p-JAK2/JAK2 and p-STAT3/STAT3 proteins were up-regulated after *DLGAP5* overexpression. Collectively, *DLGAP5* overexpression could activate the JAK2/STAT3 pathway (Figure 8).

## 3. Discussion

This study utilized bioinformatics methods (differential analysis, WGCNA, and machine learning) to identify *DLGAP5* as a potential biomarker for BC. The expression of *DLGAP5* in BC and its effects on cell proliferation, migration, invasion, and cell cycle were also validated. The KEGG enrichment analysis indicated that *DLGAP5* might be involved in the JAK/STAT pathway. Further, WB was performed to further validate the relationship between *DLGAP5* and the JAK2/STAT3 pathway, revealing the molecular mechanism of *DLGAP5* in BC.

*DLGAP5*, a member of the DLGAP protein family situated on chromosome 14 (14q22.3), is primarily distributed in the cytoplasm. It not only supports microtubule growth and stability in the spindle [11] but also acts as an oncogenic cell cycle regulator, playing a crucial role in tumor development and metastasis [12,13,14]. *DLGAP5* has been reported as an underlying prognostic factor in lung cancer, BC, clear cell renal cell carcinoma, and colorectal cancer and the overexpression of *DLGAP5* has been proved to be linked to poor prognosis in these cancers [15,16,17,18,19,20,21]. Tang et al. [22] have reached a similar conclusion by pan-cancer analysis. In addition, Zhu et al. [23] have found that *DLGAP5* was highly expressed in BC patients and also in BC cell lines (MCF-7 and MDA-MB-231). In this study, we also confirmed that *DLGAP5* is highly expressed with a poor prognosis in BC, which was validated in BC cell lines (MCF-7 and MDA-MB-231) and tissues.

Tumor development depends on cell migration, invasion, cell cycle and proliferation, among others. *DLGAP5* has been found to be up-regulated in clear cell renal cell carcinoma, and its knockdown suppressed cell viability, proliferation, migration, and invasion [17]. MicroRNA-409-5p inhibits cell proliferation and induces G2/M phase arrest and apoptosis by targeting *DLGAP5* in ovarian cancer cells [24]. Moreover, a study has been reported that *DLGAP5* knockdown inhibited BC cell proliferation [23]. In this study, the down-regulation of *DLGAP5* expression has been found to inhibit cell proliferation, migration, and invasion and arrest the cell cycle in the G2/M phase of BC cells. The opposite was true for *DLGAP5* overexpression. In addition, when the *DLGAP5* gene is knocked down, N-cadherin, MMP2, and Ki67 were degraded and the expression of E-cadherin was increased. Overexpression of *DLGAP5* had the opposite result. To sum up, *DLGAP5* could regulate cell proliferation, migration, and invasion, which might become a reliable and promising biomarker for detecting BC.

*DLGAP5* is involved in the regulatory mechanisms of cancer through multiple signaling pathways. Ke MJ et al. have reported that the knockdown of *DLGAP5* results in a significant increase in the levels of p53, p-p53, and p21 proteins, suggesting that the p53 pathway is activated in pancreatic cancer cells, which might suppress the malignant phenotype of pancreatic cancer cells [15]. Moreover, another study has proposed that *DLGAP5* is a direct target of Notch3 and acts as a tumor suppressor by modulating the cell cycle in the G2/M phase, thereby repressing tumorigenesis and cell proliferation [25]. However, the exact mechanism of *DLGAP5* in BC remained unclear. The tumor microenvironment is largely mediated by inflammatory factors [26]. The present study showed that the inhibition of *DLGAP5* resulted in a considerable reduction in *IL-6* expression, indicating that *DLGAP5* might exert an influence on BC development by affecting the inflammatory factor *IL-6* and its associated pathway signals. *IL-6* activated the JAK signaling pathway when bound to gp130. Phosphorylated JAK2 performed regulatory functions through recruiting and phosphorylating of the downstream signal STAT3. *DLGAP5* has been shown to activate the IL-6/JAK2/STAT3 signaling pathway, thereby promoting the proliferation and invasion of osteosarcoma cells [27]. In addition, it has been found that *RBMS1* may facilitate migration and invasion through the transactivation of *IL6* and the activation of the downstream signaling pathway of JAK2/STAT3 [28]. Panaxadiol has been found to inhibit the JAK2/STAT3 pathway, thus limiting the progression of pancreatic cancer [29]. Human mesenchymal stem cells derived from colorectal cancer have been shown to promote the progression of colorectal cancer through the IL-6/JAK2/STAT3 signaling pathway [30]. However, no studies have explored the relationship between *DLGAP5* and the JAK2/STAT3 pathway in BC. In this study, we found that *DLGAP5* might be involved in the JAK/STAT signaling pathway through the biosignaling method, and further verified by WB assay. The knocking down *DLGAP5* suppressed the JAK2/STAT3 signaling pathway. The overexpression of *DLGAP5* activated the JAK2/STAT3 signaling pathway. *DLGAP5* might play a carcinogenic role in BC by influencing the JAK2/STAT3 signaling pathway.

There are some limitations in this study. To begin with, only two cell lines (MCF-7 and MDA-MB-231 cell lines) were utilized to detect the role of *DLGAP5* in BC. Moreover, further in vivo validation of the function of *DLGAP5* and its relationship with the JAK2/STAT3 pathway is necessary. In conclusion, this study provides evidence that *DLGAP5* may be a potential oncogene in BC and mediates BC development through the JAK2/STAT3 pathway.

## 4. Materials and Methods

### 4.1. Data Sources and Differential Analysis

A total of six gene expression datasets of BC (GSE29431, GSE42568, GSE61304, GSE52604, GSE65194, and GSE27562) and one single-cell RNA sequencing dataset (GSE176078) were derived from the Gene Expression Omnibus database (GEO) (https://www.ncbi.nlm.nih.gov/geo/ (accessed on 27 February 2023)). These selected datasets included the following samples: GSE29431: 12 normal and 54 BC samples; GSE42568: 17 normal and 104 BC samples; GSE61304: 4 normal and 62 BC samples; GSE52604: 20 normal and 35 BC samples; GSE65194: 11 normal and 167 BC samples; GSE27562: 31 normal and 131 BC samples; GSE176078: 26 BC samples. Furthermore, gene expression matrix and clinical information data of BC and paracancer tissues were acquired from TCGA database (https://portal.gdc.cancer.gov/repository (accessed on 28 February 2023)), which included a total of 112 normal samples and 1076 tumor samples. Differential analysis was performed on GSE42568, GSE61304, and GSE29431 through the “limma” package of R (version 3.54.2). |log_2_FC| > 1.5 and *p* < 0.05 were considered as the screening thresholds. The volcano plot of all Differential Expressed Genes (DEGs) and the cluster heatmap of top 50 up-regulated and down-regulated genes were plotted by “ggplot2” (version 3.4.2) and “pheatmap” package (version 1.0.12) of R, separately.

### 4.2. WGCNA Construction and Module Identification

“WGCNA” package of R software (version 1.72-1) was used to implement WGCNA, a method for constructing co-expression networks and identifying gene modules highly correlated with specific phenotypes or traits [31]. To begin with, a clustering analysis was performed on the sample to detect potential outliers. Next, the “pickSoftThreshold” function of R determined the soft-thresholding power β (β = 6, R^2^ = 0.9) and suggested co-expression similarities for computing the adjacency relationship. Modules were detected using hierarchical clustering and dynamic tree-cutting functions. Finally, gene significance (GS) and module membership (MM) were calculated to correlate modules with clinical traits. The information on the corresponding module genes was extracted for further analyses.

### 4.3. Protein–Protein Interaction Networks (PPI) Construction

The DEGs were intersected with the modular genes screened by WGCNA and the interacting genes were visualized using Sangerbox (http://sangerbox.com/ (accessed on 17 March 2023)) [32]. Then, the PPI networks for the intersecting genes were constructed through the STRING database.

### 4.4. Key Gene Screening

The MCODE (Cytoscape’s plug-in, Version 2.0.2) was utilized to choose the genes. To further screen key genes, machine learning techniques (LASSO, RF, SVM-RFE, and XGBoost) were also applied. RF and LASSO logistic regression analyses were carried out using the “glmnet” (version 4.1-7) and “randomForest” (version 4.7-1.1) packages. SVM-RFE was a support vector machine- learning-based approach to finding the best variables by removing the feature vectors generated by SVM and further identifying the relevant values of these biomarkers in BC by the “e1071” (version 1.7-13) and “caret” (version 6.0-94) packages. Extreme gradient boosting (XGBoost) was a commonly supervised integrated-learning algorithm which was performed using the “xgboost” package (Version 1.7.5.1) to rank the importance of characteristic genes, and to plot the ROC and AUC to assess the diagnostic value of genes.

### 4.5. Survival, Differential Expression and Diagnostic Accuracy Analyses of Key Genes

The Kaplan–Meier plotter database was used for the survival analysis of signature genes in BC to further determine their clinical value. Afterwards, the expression distribution and diagnostic accuracy of key genes were analyzed within the training groups (GSE29431 and GSE42568) and test groups (GSE52604, GSE65194, and GSE27562). The boxplots plotted by using the R package “ggpubr” showed the differential expression distribution of key genes between the control and BC groups. The diagnostic accuracy of key genes associated with BC was evaluated by AUC scoring of ROC curves through the R package “pROC”. GEPIA database was utilized to validate the expression of key genes [33].

The expression of key genes in breast and BC tissues was verified by the HPA database. Moreover, the seven BC samples and seven para-cancer tissue samples were collected from Shaanxi Cancer Hospital for immunohistochemistry assays. The clinical information of the patients was complete and informed consent was obtained from all patients prior to sample collection.

### 4.6. Analysis of the Single-Cell RNA Sequencing Dataset

The GSE176078 dataset, containing the BC samples, was downloaded from GEO database and analyzed using the R packages, “Seurat” [34] and “SingleR” [35]. At least three cells expressed features and nFeature > 200, nFeature RNA < 4000, and mitochondrial gene <10% were retained. The FindNeighbors and FindClusters functions were clustered in the cells. The FindAllMarkers function was used to identify markers and annotated cells according to these markers (min.pct = 0.5, log_2_FC threshold = 0.5). The FindMarkers function was used to analyze the difference between two specified groups of cells (|avg_log_2_FC| > 1, *p*_val_adj < 0.05). Cell group annotation was made through CellMarker 2.0 website (http://117.50.127.228/CellMarker/ (accessed on 15 March 2023)) [36].

### 4.7. Enrichment Analysis of DLGAP5

The *DLGAP5*-binding proteins were retrieved at the STRING website. GO and KEGG enrichment analyses of these genes were performed using the DAVID database.

### 4.8. Cell Culture and Stable Cell Line Construction

The lentivirus-mediated *DLGAP5* overexpression vector, small interfering RNA (siRNA), and negative control vector were designed and synthesized by GenePharma Co. (Shanghai, China) (interfering sequence information in Supplement Table S1). They were transfected into MCF-7 and MDA-MB-231 cells (purchased from Procell Life Sciences Co., Wuhan, China), respectively. And then they were incubated with purinomycin (2.5 μg/mL) to produce stable transfected cell lines. qRT-PCR and WB were used to detect overexpression and knockdown efficiency.

### 4.9. RNA Extraction and qRT-PCR

Total RNA for cDNA synthesis was isolated from cells by Trizol reagent (Invitrogen, Carlsbad, CA, USA). The samples were assayed for quality by the NanoDrop One instrument. Total RNA was reverse transcribed to cDNA based on mRNA reverse transcription kit (Takara, Kyoto, Japan) and gene amplification thermal cycler. And the reverse transcription reaction conditions were 37 °C for 15 min, 85 °C for 5 s, and 4 °C for ∞. The cDNA, Mix solution and forward and reverse primers were mixed to perform qRT-PCR. GAPDH as housekeeping gene could regulate relative mRNA expression. Reaction procedure: 95 °C for 30 s; denaturation annealing extension for 45 cycles, 95 °C for 10 s, 60 °C for 30 s, 72 °C for 10 s. Relative gene expression levels in cells were calculated using 2^−∆∆CT^. The primer sequences are provided in Supplement Table S2.

### 4.10. Western Blot

Protein lysis solution (containing PMSF) and extraction buffer were added to the cells to extract total protein and their concentration was determined by the BCA kit. The total protein sample was separated by SDS-PAGE and then transferred to the polyvinylidene fluoride membranes. The primary antibody was incubated with the membranes at 4 °C for the night. After TBST washing, the film and the secondary antibody were incubated at room temperature for two hours. The luminescent solution was added and left to stand for two minutes protected from light after TBST washing three times. AllCap software (Version 1.0)was used to adjust the focus gap and take photos.

### 4.11. CCK8

Cell proliferation was determined using the CCK-8 kit according to the manufacturer’s instructions. The cell concentration was adjusted to 3 × 10^4^ cells/mL and evenly spread into the 96-well plate at 100 μL/well. After continuous incubation in an incubator for 24, 48, and 72 h, 10 μL CCK-8 mix was added to each well and incubated for an additional 2 h at 37 °C. Then, the absorbance at 450 nm was measured using an enzyme microplate reader.

### 4.12. Colony Formation Assay

The cell volumes were adjusted to 500 cells/mL, 250 cells/mL, and 125 cells/mL, respectively, and 2 mL was inoculated into the corresponding 6-well plates. The six-well plates were removed to observe the cells and fluid was exchanged every 3–4 days. When the number of cells in each population reached more than 50, chromatography was performed. The cells were washed with PBS, fixed with 600 μL of 4% paraformaldehyde for 15 min, and stained with 0.1% crystal violet.

### 4.13. Wound-Healing Assay

The cell migration was assessed by performing wound-healing assay. MCF-7 and MDA-MB-231 cells with different treatment were seeded into 6-well plates and cultured in DMEM with 10% FBS (Procell, Wuhan, China, 164210-500). After cells reached 80% confluence, a line wound was scratched by a 200 μL tip and the detached cells were removed by washing with PBS (Procell, Wuhan, China, PB180327). Subsequently, the cells were cultured in serum-free DMEM. The wounds were observed at 0 h, 24 h, and 48 h. The wound-healing rate was calculated according to the following formula: Wound-healing rate = (X − Y)/X, X: the wound width at 0 h, Y: the wound width at 24 h or 48 h.

### 4.14. Transwell Assay

Cell invasion and migration were tested using a 24-well Transwell plate. The Matrigel basal gel was diluted at 4 °C, then dripped into the underclock of the chamber, with a gun head to remove the bubble, and then put back into the cultivation box for 2–4 h (without this step in the migration experiments). Then, 600 μL RPMI 1640 medium containing 10% FBS was added into the lower 24-well chamber, and 100–150 μL cell suspension (2 × 10^5^ cells/mL) was added into the upper chamber, and continued cultivation for 24 h. The lower surface was soaked in 70% methanol solution, fixed for 20 min, and stained with 0.25% crystal violet for 20 min. Five visual fields in and around the center were randomly selected under the microscope to be photographed. ImageJ software (Version 1.53t) was used to calculate the number of invasions and migrations, and the mean value was taken.

### 4.15. Flow Cytometry

The cells were digested with EDTA-free pancreatic enzymes and then collected. PBS was added to the precipitate to re-suspend the cells, which were centrifuged at 472× *g* for 5 min. After centrifugation, 0.5 mL staining solution of cell cycle (PI:RNase = 9:1, Nanjing KeyGen Biotech. Co., Ltd., Nanjing, China) was added, and the reaction was conducted at room temperature for 1 h away from light. The cell cycle was then measured by flow cytometry. The cell cycle results were processed with Modifit software (Version 5.0) and the graphs were plotted with GraphPad software (Version 8.0).

### 4.16. Data Analysis

For bioinformatics validation, all gene expression data were naturalized by log_2_ transformation. The connection between the *DLGAP5* expression and the target was evaluated using Spearman’s or Pearson’s test. All survival analyses in this study were based on the Kaplan–Meier curves. Depending on whether the samples were paired or not, a paired *t*-test or *t*-test was used to compare *DLGAP5* expression levels between groups or between tumors and normal tissues. Significance was defined as *p* < 0.05. All statistical analyses were processed by R software (version 4.2.1). For molecular biology verification, all statistical calculations were carried out using GraphPad Prism (Version 8.0, GraphPad Software Inc., San Diego, CA, USA). The data reported are the SD ± average. Double-tailed Student’s *t*-tests were performed to determine statistically significant differences. If the *p* value was <0.05, the difference was defined as of statistical significance.

## 5. Conclusions

This study identified *DLGAP5* as a potential biomarker for BC and it was found to play a crucial role in regulating the processes of proliferation, migration, invasion, and cell cycle of BC cell lines. Furthermore, it was observed that *DLGAP5* also regulated the activity of the JAK2/STAT3 signaling pathway involved in cancer progression and development. These findings highlighted the significance of *DLGAP5* in BC and provided valuable insights into its potential as a therapeutic target and diagnostic biomarker.

## Data Availability

The datasets used or analyzed during the current study are available from the corresponding author upon reasonable request.

## Supplementary Materials

The following supporting information can be downloaded at: https://www.mdpi.com/article/10.3390/ijms242115819/s1.
