# Supplementary material for: DLGAP5 Regulates the Proliferation, Migration, Invasion, and Cell Cycle of Breast Cancer Cells via the JAK2/STAT3 Signaling Axis
Source: Int J Mol Sci. 2023 Oct 31;24(21):15819. doi: 10.3390/ijms242115819 (PMC10647495; doi:10.3390/ijms242115819)
Supplement: Supplementary file 1 [file ijms-24-15819-s001.zip › supplementary table.pdf]

Supplementary Table S1. Interferes with sequence information.

Gene name	Sequence	
	Sense（5'-3'）	Antisense（5'-3'）
DLGAP5-Negative	UUCUCCGAACGUGUCACGUTT	ACGUGACACGUUCGGAGAATT
DLGAP5-si-RNA#1	GGAGCAGACUAAGAUUGAUTT	AUCAAUCUUAGUCUGCUCCTT
DLGAP5-si-RNA#2	CCUAUCAAGUAACACCUAUTT	AUAGGUGUUACUUGAUAGGTT
DLGAP5-si-RNA#3	GCAAUGAGAGAGAGAAUUATT	UAAUUCUCUCUCUCAUUGCTT

Supplementary Table S2. Primer sequence information.

Gene name	Primer	Direction	Sequence
DLGAP5	H-*DLGAP5*-F	5'-3'	ATTCGCACAGCAGTTGGTCA
	H-*DLGAP5*-R	5'-3'	GCATCTTCTATGCTGCTCCTGC
GAPDH	H-*GAPDH*-F	5'-3'	GGAGCGAGATCCCTCCAAAAT
	H-*GAPDH*-R	5'-3'	GGCTGTTGTCATACTTCTCATGG
Cyclin D1	H-*Cyclin D1*-F	5'-3'	GTCCTACTTCAAATGTGTGCAG
	H-*Cyclin D1*-R	5'-3'	GGGATGGTCTCCTTCATCTTAG
Ki67	H-*Ki67*-F	5'-3'	ACTCTCCACAGTCAGACCCA
	H-*Ki67*-R	5'-3'	TGCACGCTAAGAGTTCTCCC
IL-6	H-*IL-6*-F	5'-3'	TCCTTCTCCACAAGCGCC
	H-*IL-6*-R	5'-3'	GATGCCGTCGAGGATGTACC

Supplementary Table S3. Results of GO enrichment analysis of *DLGAP5* related genes.

Type	Term	Count	PValue	Fold Enrichment	Bonferroni	Benjamini	FDR
MF	GO:0005515-protein binding	276	7.22E-29	1.40	2.83E-26	2.83E-26	2.44E-26
MF	GO:0019901-protein kinase binding	47	4.27E-22	5.84	1.67E-19	8.37E-20	7.22E-20
MF	GO:0016538-cyclin-dependent protein serine/threonine kinase regulator activity	17	1.86E-21	35.16	7.28E-19	2.43E-19	2.09E-19
MF	GO:0008017-microtubule binding	34	1.21E-20	8.32	4.74E-18	1.19E-18	1.02E-18
MF	GO:0005524-ATP binding	77	2.74E-20	3.21	1.07E-17	2.15E-18	1.85E-18
MF	GO:0003777-microtubule motor activity	17	6.01E-16	18.17	2.18E-13	3.93E-14	3.39E-14
MF	GO:0003682-chromatin binding	35	7.29E-14	4.80	2.86E-11	4.08E-12	3.52E-12
MF	GO:0017056-structural constituent of nuclear pore	12	2.87E-13	26.53	1.12E-10	1.41E-11	1.21E-11
MF	GO:0004712-protein serine/threonine/tyrosine kinase activity	32	2.34E-12	4.66	9.17E-10	1.02E-10	8.78E-11
MF	GO:0004674-protein serine/threonine kinase activity	30	5.15E-12	4.84	2.02E-09	2.02E-10	1.74E-10
CC	GO:0005654-nucleoplasm	209	7.77E-83	3.67	2.69E-80	2.69E-80	2.04E-80
CC	GO:0005634-nucleus	218	7.10E-57	2.53	2.45E-54	1.23E-54	9.29E-55
CC	GO:0005829-cytosol	204	7.38E-52	2.57	2.55E-49	8.51E-50	6.44E-50
CC	GO:0000776-kinetochore	40	1.49E-37	17.95	5.16E-35	1.29E-35	9.77E-36
CC	GO:0005813-centrosome	60	3.48E-34	7.48	1.20E-31	2.41E-32	1.82E-32
CC	GO:0005819-spindle	34	4.13E-30	16.09	1.43E-27	2.38E-28	1.80E-28
CC	GO:0005680-anaphase-promoting complex	19	1.21E-29	57.45	4.19E-27	5.98E-28	4.53E-28
CC	GO:0000775-chromosome, centromeric region	25	3.21E-28	27.17	1.11E-25	1.39E-26	1.05E-26
CC	GO:0072686-mitotic spindle	32	3.84E-28	15.90	1.33E-25	1.48E-26	1.12E-26
CC	GO:0005694-chromosome	39	1.40E-27	10.64	4.85E-25	4.85E-26	3.67E-26
BP	GO:0051301-cell division	119	5.01E-126	20.15	9.64E-123	9.64E-123	8.79E-123
BP	GO:0007049-cell cycle	59	1.17E-42	10.77	2.24E-39	1.12E-39	1.02E-39
BP	GO:0000278-mitotic cell cycle	42	3.12E-40	18.50	6.00E-37	2.00E-37	1.82E-37
BP	GO:0000079-regulation of cyclin-dependent protein serine/threonine kinase activity	27	4.85E-33	32.00	9.34E-30	2.33E-30	2.13E-30

BP	GO:0007346-regulation of mitotic cell cycle	28	2.43E-32	28.08	4.68E-29	9.36E-30	8.53E-30
BP	GO:0007059-chromosome segregation	27	3.43E-28	22.56	6.60E-25	1.10E-25	1.00E-25
BP	GO:0000070-mitotic sister chromatid segregation	21	1.49E-27	38.02	2.86E-24	4.08E-25	3.72E-25
BP	GO:0051445-regulation of meiotic cell cycle	17	2.72E-26	55.40	5.23E-23	6.54E-24	5.96E-24
BP	GO:0007052-mitotic spindle organization	23	1.10E-25	26.30	2.11E-22	2.35E-23	2.14E-23
BP	GO:0007094-mitotic spindle assembly checkpoint	18	3.92E-23	36.66	7.54E-20	7.54E-21	6.87E-21

Supplementary Table S4. Enrichment results of KEGG pathway of *DLGAP5* related genes.

Term	Count	PValue	Fold Enrichment	Bonferroni	Benjamini	FDR
hsa04110:Cell cycle	86	1.23E-114	28.49	2.68E-112	2.68E-112	2.05E-112
hsa04114:Oocyte meiosis	45	4.70E-40	14.45	1.02E-37	5.10E-38	3.90E-38
hsa04914:Progesterone-mediated oocyte maturation	39	1.50E-36	16.09	3.25E-34	1.08E-34	8.29E-35
hsa05166:Human T-cell leukemia virus 1 infection	48	1.48E-32	9.10	3.21E-30	8.04E-31	6.15E-31
hsa04218:Cellular senescence	40	6.57E-30	10.79	1.43E-27	2.85E-28	2.18E-28
hsa04115:p53 signaling pathway	23	4.30E-19	13.26	9.34E-17	1.56E-17	1.19E-17
hsa05203:Viral carcinogenesis	34	5.05E-19	7.01	1.10E-16	1.57E-17	1.20E-17
hsa03013:Nucleocytoplasmic transport	24	3.13E-16	9.35	7.23E-14	8.49E-15	6.49E-15
hsa04120:Ubiquitin mediated proteolysis	25	1.86E-14	7.41	4.02E-12	4.48E-13	3.43E-13
hsa05220:Chronic myeloid leukemia	19	9.13E-14	10.52	1.98E-11	1.98E-12	1.52E-12
hsa05222:Small cell lung cancer	20	2.64E-13	9.15	5.73E-11	5.21E-12	3.98E-12
hsa05200:Pathways in cancer	42	7.36E-12	3.33	1.60E-09	1.33E-10	1.02E-10
hsa05169:Epstein-Barr virus infection	24	3.03E-10	5.00	6.57E-08	5.05E-09	3.86E-09
hsa03030:DNA replication	12	3.49E-10	14.03	7.57E-08	5.41E-09	4.14E-09
hsa05223:Non-small cell lung cancer	15	9.58E-10	8.77	2.08E-07	1.39E-08	1.06E-08

hsa04068:FoxO signaling pathway	18	9.88E-09	5.78	2.14E-06	1.34E-07	1.02E-07
hsa05165:Human papillomavirus infection	28	1.41E-08	3.56	3.05E-06	1.80E-07	1.37E-07
hsa05219:Bladder cancer	11	2.57E-08	11.29	5.57E-06	3.09E-07	2.37E-07
hsa05215:Prostate cancer	15	5.34E-08	6.51	1.16E-05	6.10E-07	4.67E-07
hsa01522:Endocrine resistance	15	6.10E-08	6.44	1.32E-05	6.62E-07	5.06E-07
hsa05218:Melanoma	13	9.65E-08	7.60	2.09E-05	9.97E-07	7.62E-07
hsa05214:Glioma	13	1.54E-07	7.29	3.34E-05	1.52E-06	1.16E-06
hsa05212:Pancreatic cancer	13	1.79E-07	7.20	3.89E-05	1.69E-06	1.29E-06
hsa05161:Hepatitis B	18	2.38E-07	4.68	5.17E-05	2.15E-06	1.65E-06
hsa05226:Gastric cancer	17	3.99E-07	4.80	8.66E-05	3.46E-06	2.65E-06
hsa05207:Chemical carcinogenesis - receptor activation	20	5.45E-07	3.97	1.18E-04	4.54E-06	3.48E-06
hsa05162:Measles	16	8.76E-07	4.84	1.90E-04	7.04E-06	5.38E-06
hsa05206:MicroRNAs in cancer	24	9.90E-07	3.26	2.15E-04	7.67E-06	5.87E-06
hsa05224:Breast cancer	16	1.79E-06	4.58	3.89E-04	1.31E-05	1.00E-05
hsa05216:Thyroid cancer	9	1.81E-06	10.23	3.93E-04	1.31E-05	1.00E-05
hsa05213:Endometrial cancer	10	7.50E-06	7.25	0.001627	5.25E-05	4.02E-05
hsa04934:Cushing syndrome	15	1.66E-05	4.07	0.003596	1.13E-04	8.61E-05
hsa04919:Thyroid hormone signaling pathway	13	2.67E-05	4.52	0.005775	1.76E-04	1.34E-04
hsa05210:Colorectal cancer	11	3.22E-05	5.38	0.006959	2.05E-04	1.57E-04
hsa04340:Hedgehog signaling pathway	9	4.54E-05	6.76	0.009804	2.81E-04	2.15E-04
hsa05202:Transcriptional misregulation in cancer	16	4.90E-05	3.49	0.010572	2.95E-04	2.26E-04
hsa05163:Human cytomegalovirus infection	17	7.97E-05	3.18	0.01715	4.68E-04	3.58E-04
hsa05160:Hepatitis C	14	8.30E-05	3.75	0.017858	4.74E-04	3.63E-04
hsa03420:Nucleotide excision repair	8	1.05E-04	7.16	0.022447	5.82E-04	4.45E-04
hsa05225:Hepatocellular carcinoma	14	1.64E-04	3.51	0.035067	8.86E-04	6.78E-04
hsa04012:ErbB signaling pathway	10	1.67E-04	4.95	0.035685	8.86E-04	6.78E-04

hsa04151:PI3K-Akt signaling pathway	21	2.54E-04	2.50	0.053703	0.001314	0.001005
hsa05205:Proteoglycans in cancer	15	3.43E-04	3.08	0.071685	0.00173	0.001323
hsa05167:Kaposi sarcoma-associated herpesvirus infection	14	6.67E-04	3.04	0.134721	0.003227	0.002468
hsa05217:Basal cell carcinoma	8	6.69E-04	5.34	0.135192	0.003227	0.002468
hsa05221:Acute myeloid leukemia	8	9.71E-04	5.02	0.190145	0.004583	0.003506
hsa04917:Prolactin signaling pathway	8	0.001262	4.81	0.239635	0.005825	0.004456
hsa04210:Apoptosis	11	0.001392	3.40	0.260804	0.006291	0.004813
hsa03430:Mismatch repair	5	0.001886	9.15	0.336092	0.008352	0.006389
hsa05014:Amyotrophic lateral sclerosis	19	0.002381	2.20	0.403907	0.010335	0.007906
hsa03250:Viral life cycle - HIV-1	7	0.003588	4.68	0.541563	0.015265	0.011678
hsa04390:Hippo signaling pathway	11	0.003999	2.95	0.580871	0.016689	0.012767
hsa04540:Gap junction	8	0.00468	3.83	0.638701	0.019163	0.01466
hsa01524:Platinum drug resistance	7	0.007404	4.03	0.800638	0.029753	0.02276
hsa05170:Human immunodeficiency virus 1 infection	12	0.011589	2.38	0.920309	0.045726	0.034979
hsa04310:Wnt signaling pathway	10	0.019223	2.48	0.985182	0.074487	0.056981
hsa05164:Influenza A	10	0.019885	2.46	0.987202	0.075701	0.05791
hsa04010:MAPK signaling pathway	14	0.022014	2.00	0.992017	0.082364	0.063007
hsa04935:Growth hormone synthesis, secretion and action	8	0.023426	2.81	0.994165	0.086158	0.065909
hsa04350:TGF-beta signaling pathway	7	0.02475	3.10	0.995654	0.089514	0.068476
hsa05034:Alcoholism	10	0.033043	2.25	0.999319	0.117546	0.08992
hsa04728:Dopaminergic synapse	8	0.036758	2.55	0.999704	0.128653	0.098417
hsa04062:Chemokine signaling pathway	10	0.038024	2.19	0.999778	0.130971	0.10019
hsa03410:Base excision repair	4	0.042239	5.10	0.999914	0.143177	0.109527
hsa04630:JAK-STAT signaling pathway	9	0.043006	2.28	0.999928	0.143177	0.109527
hsa04910:Insulin signaling pathway	8	0.043547	2.46	0.999936	0.143177	0.109527
hsa05418:Fluid shear stress and atherosclerosis	8	0.046475	2.42	0.999967	0.148929	0.113927

hsa04530:Tight junction	9	0.046669	2.24	0.999969	0.148929	0.113927
hsa04213:Longevity regulating pathway - multiple species	5	0.058505	3.39	0.999998	0.183994	0.140751
hsa03440:Homologous recombination	4	0.072078	4.11	1	0.221394	0.169361
hsa04921:Oxytocin signaling pathway	8	0.072438	2.19	1	0.221394	0.169361
hsa04720:Long-term potentiation	5	0.073538	3.14	1	0.221636	0.169547
hsa05031:Amphetamine addiction	5	0.080051	3.05	1	0.237959	0.182033
hsa04926:Relaxin signaling pathway	7	0.085001	2.28	1	0.246366	0.188464
hsa04962:Vasopressin-regulated water reabsorption	4	0.08515	3.83	1	0.246366	0.188464
hsa04933:AGE-RAGE signaling pathway in diabetic complications	6	0.087817	2.52	1	0.250741	0.191811
hsa04064:NF-kappa B signaling pathway	6	0.099701	2.43	1	0.277374	0.212185
hsa04660:T cell receptor signaling pathway	6	0.099701	2.43	1	0.277374	0.212185

Supplementary Table S5. Gene list of JAK-STAT pathway.

KEGG pathway name	Gene list
Cell cycle	*IL2, IL4, IL7, IL9, IL15, IL21, TSLP, IL2RA, IL2RB, IL2RG, IL4R, IL7R, IL9R, IL15RA, IL21R, CRLF2, IL3, IL5, CSF2, IL3RA, IL5RA, CSF2RA, CSF2RB, IL6, IL11, IL13, IL31, OSM, LIF, CNTF, CTF1, CLCF1, IL6R, IL6ST, IL11RA, IL13RA1. IL13RA2, IL27RA, IL31RA, OSMR, LIFR, CNTFR, IL10, IL19, IL22, IL20, IL24, IL26, IL10RA, IL10RB, IL20RA, IL20RB. IL22RA1, IL22RA2, IL12A, IL12B, IL23A, IL12RB1, IL12RB2, IL23R, EPO, GH1,PRL, THPO. CSF3. LEP. C19orf43. EPOR. GHR. PRLR, MPL, CSF3R, LEPR, IFNA7, IFNB1, IFNE, IFNK, IFNL1, IFNW1, IFNAR1, IFNAR2, IFNLR1, IFNG, IFNGR1, IFNGR2, EGF, PDGFA, PDGFB, EGFR, PDGFRA, PDGFRB, JAK1, JAK2, JAK3, TYK2, STAT1, STAT2, STAT3, STAT4, STAT5A, STAT5B, STAT6, IRF9, CISH, SOCS1, SOCS2, SOCS3, SOCS4, SOCS5, BCL2, BCL2L1, MYC, CCND1, CCND2, CCND3, CDKN1A, AOX1, GFAPSTAM2, PTPN2, PTPN6, PIAS1, PIAS2, PIAS3, PIAS4, FHL1, PTPN11, GRB2, SOS2, HRAS, IL27A, CTF2, DOME, IL28, SOCS6_7, MCL1, PIM1, KAT3, RAF1, PIK3CA_B_D, PIK3R1_2_3, AKT, MTOR*

Supplementary Table S6 JAK-STAT pathway-related genes significantly correlated with *DLGAP5* ($|R| > 0.3$, $P < 0.05$)

Gene	Gene	Cor	P
DLGAP5	*IL21*	0.34	1.31E-32
DLGAP5	*TSLP*	-0.30	8.70E-26
DLGAP5	*IL2RA*	0.36	1.43E-36
DLGAP5	*IL21R*	0.43	2.20E-16
DLGAP5	*CTF1*	-0.31	1.02E-27
DLGAP5	*IL12RB2*	0.30	5.97E-26
DLGAP5	*IFNB1*	0.34	1.19E-32
DLGAP5	*IFNL1*	0.39	9.24E-43
DLGAP5	*STAT1*	0.51	1.72E-77
DLGAP5	*SOCS2*	-0.33	8.38E-30
DLGAP5	*SOCS4*	0.36	2.59E-36
DLGAP5	*GFAP*	-0.37	2.42E-38
DLGAP5	*PTPN2*	0.39	2.05E-43
DLGAP5	*FHL1*	-0.31	5.89E-27
DLGAP5	*GRB2*	0.36	1.80E-35
DLGAP5	*RAF1*	0.39	4.93E-42
