# Supplementary figures and images for: DLGAP5 Regulates the Proliferation, Migration, Invasion, and Cell Cycle of Breast Cancer Cells via the JAK2/STAT3 Signaling Axis
Source: Int J Mol Sci. 2023 Oct 31;24(21):15819. doi: 10.3390/ijms242115819 (PMC10647495; doi:10.3390/ijms242115819)

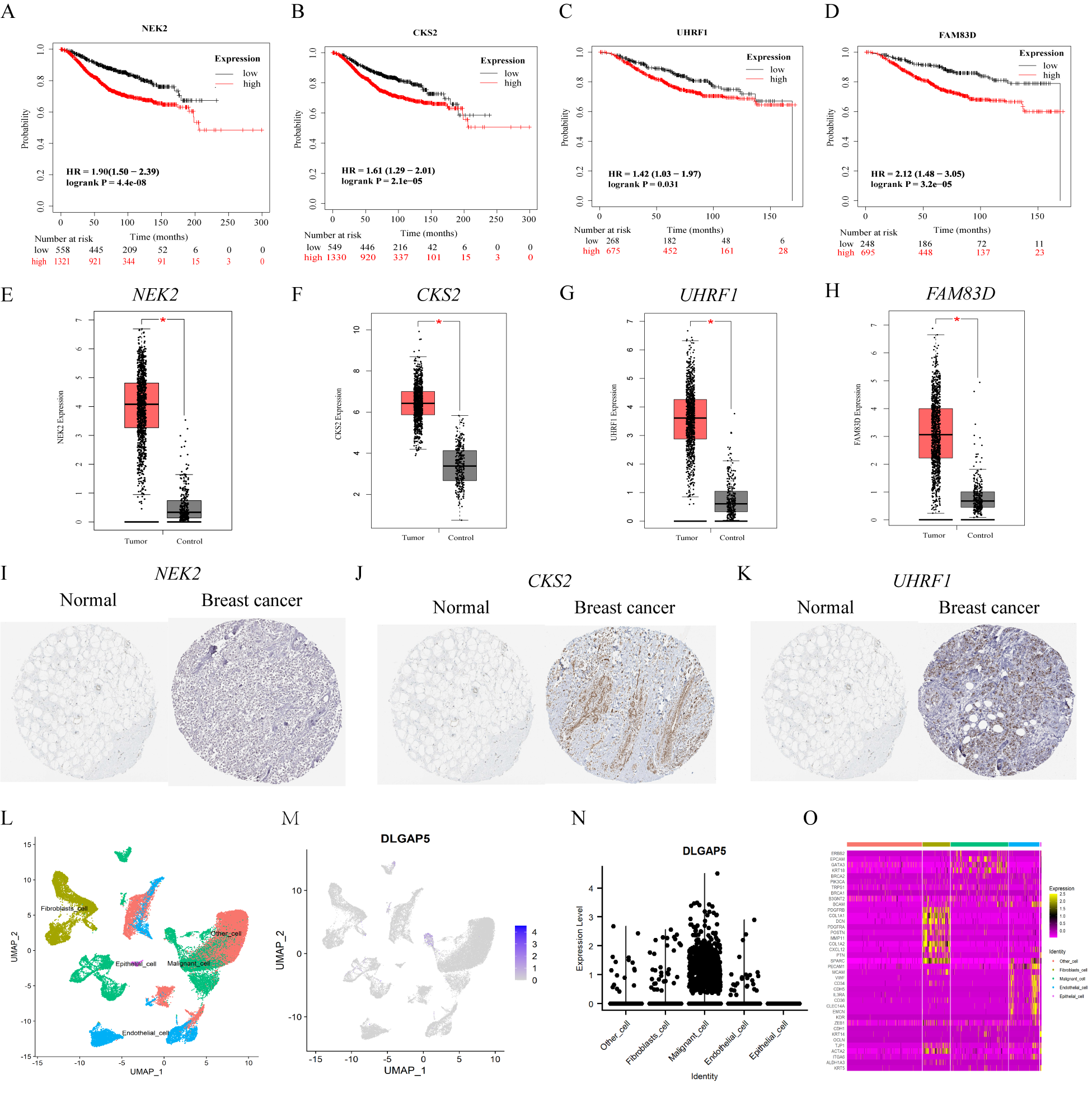

Supplement: Supplementary file 1 [file ijms-24-15819-s001.zip › Figure S1.tif]

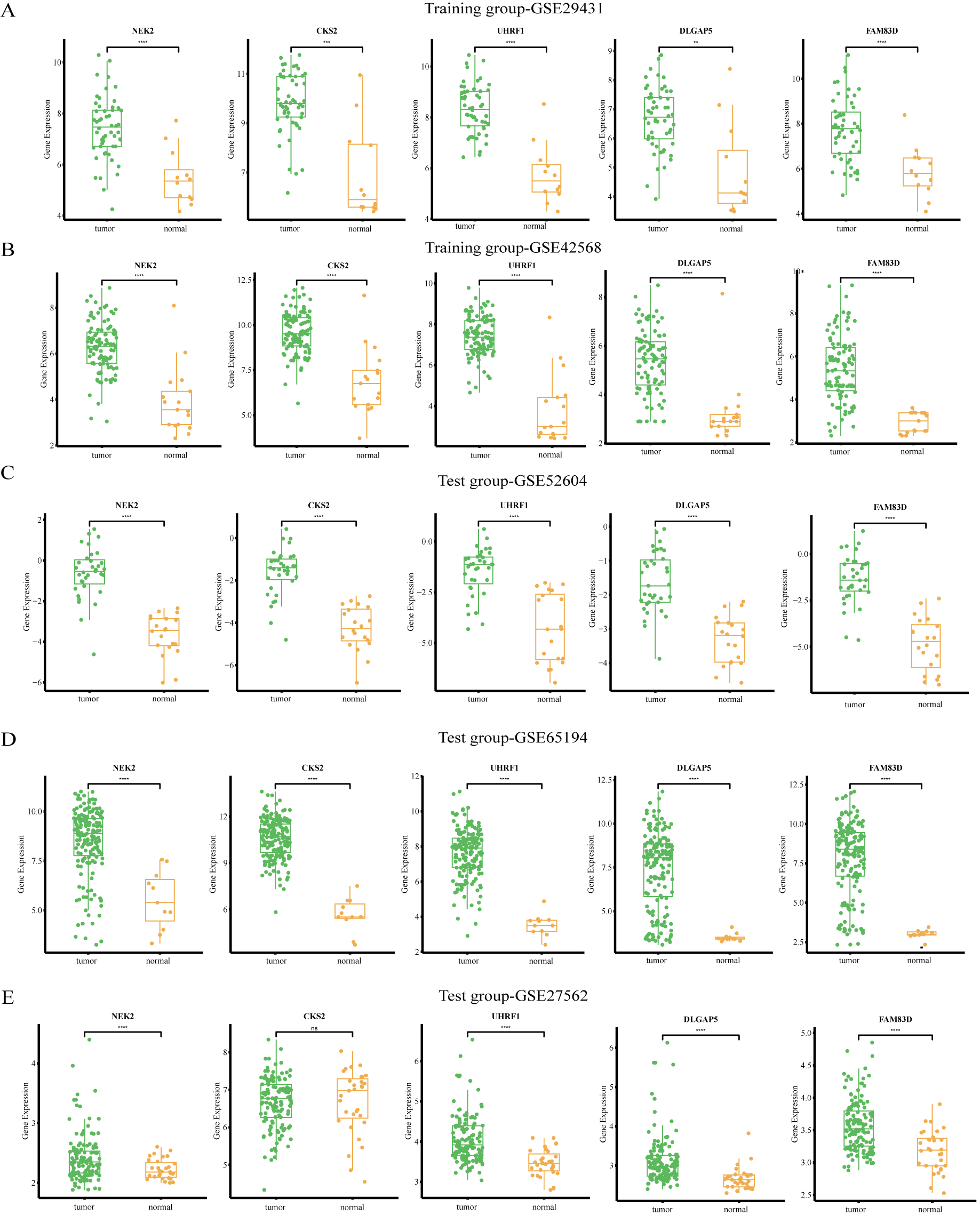

Supplement: Supplementary file 1 [file ijms-24-15819-s001.zip › Figure S2.tif]

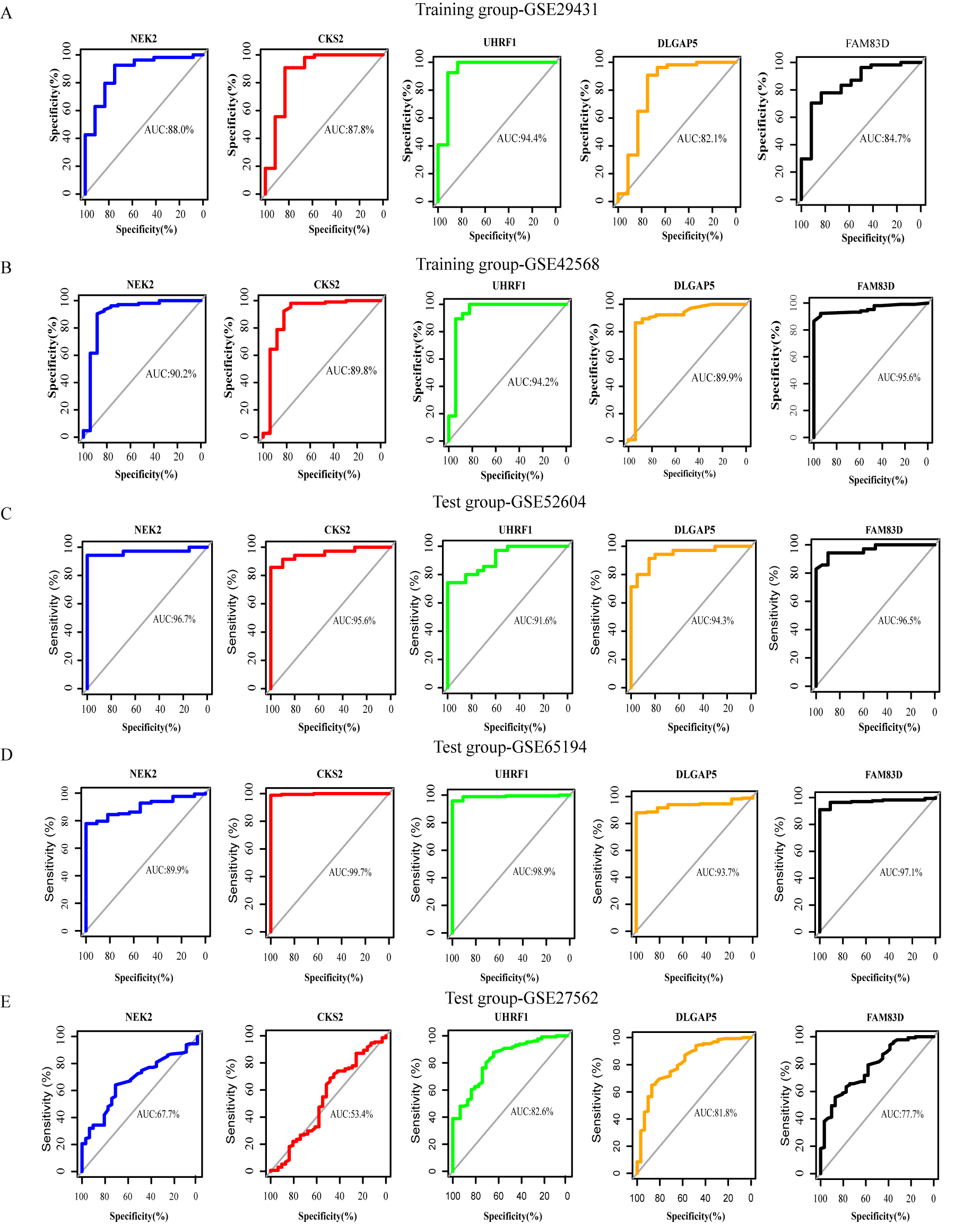

Supplement: Supplementary file 1 [file ijms-24-15819-s001.zip › Figure S3.tif]

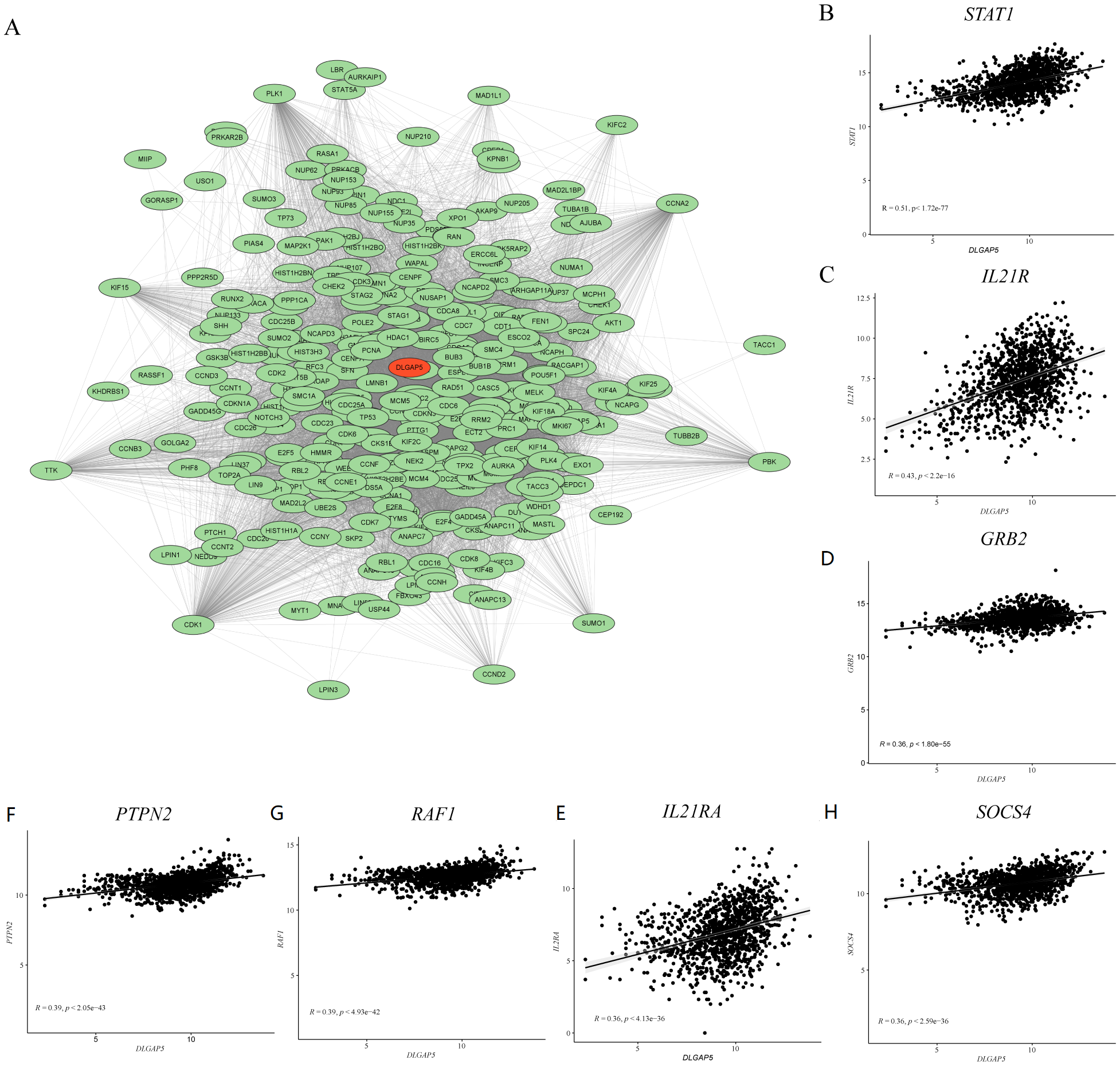

Supplement: Supplementary file 1 [file ijms-24-15819-s001.zip › Figure S4.tif]
